# Osteopetrosis with Typical Radiological Findings: A Report of a Rare Case

**DOI:** 10.4314/ejhs.v34i2.8

**Published:** 2024-03

**Authors:** Bethlehem Tesfasilassie Kibrom, Tesfahunegn Hailemariam Feleke, Wubineh Admasu, Frehiwot Tsegaye, Samuel Sisay Hailu

**Affiliations:** 1 Addis Ababa University, College of Health Sciences, School of Medicine, Addis Ababa, Ethiopia; 2 Addis Ababa University, College of Health Sciences, School of Medicine, Department of Radiology, Addis Ababa, Ethiopia

**Keywords:** Osteopetrosis, Sclerosing skeletal dysplasia, Pathologic fracture, Cranial neuropathy

## Abstract

**Background:**

Osteopetrosis is a rare inherited disease caused by a lack of osteoclastic bone resorption, resulting in increased bone mass with insufficient mechanical strength. Patients usually present with complications such as pathologic fractures, cranial neuropathies, and bone marrow failure. Diagnosis is based on suggestive clinical and imaging findings, with genetic testing to confirm diagnosis and identify the subtype.

**Case Presentation:**

The patient is an eight-year-old girl who came to our hospital with complaints of bilateral arm swelling and visual disturbances for four years and a history of fracture of the left lower extremity two years before presentation. Physical examination revealed nontender bilateral arm swelling and a shorter left leg. The laboratory tests were within normal limits. A skeletal radiograph showed typical radiographic features of osteopetrosis.

**Conclusion:**

It is important to consider osteopetrosis in patients presenting with pathologic fractures and cranial neuropathies. Typical imaging findings can provide rapid diagnosis in severe cases.

## Introduction

Osteopetrosis, or marble bone disease, is a group of rare inherited metabolic bone diseases characterized by abnormally high bone mineral density and generalized osteosclerosis caused by osteoclast failure (1-4). Although osteoclast dysfunction results in high bone mineral density, the affected bones are unusually brittle. There are four forms of osteopetrosis, including the malignant autosomal recessive form (ARO), the intermediate autosomal recessive form (IAO), and the two subclassifications of autosomal dominant osteopetrosis (ADO), types I and II (1). The incidence of the autosomal recessive form is about 1 in 200,000-250,000, making it far less common than the autosomal dominant form, which is 1 in 20,000 (1, 2, 4).

The clinical symptoms, age of onset, and prognosis (from mild to severe) differ depending on the type of osteopetrosis (2, 3). However, signs and symptoms may include fractures, skeletal deformity, and dental abnormalities. Further complications include cranial neuropathies and bone marrow failure due to narrow cranial foramina and marrow space compromise, respectively, and interference with mineral homeostasis such as calcium and phosphorus (2). The optic nerve is the most commonly affected nerve, followed by the auditory, trigeminal, and facial nerves (1). In this report, we present a case of osteopetrosis with typical imaging features in an 8-year-old girl with progressive vision loss and a past history of fracture.

## Case Presentation

An 8-year-old female child was brought to our hospital with a complaint of bilateral arm swelling and progressive vision loss of 4 years duration. She had a history of a left leg fracture two years before presentation. There was no other relevant past self or family medical history. Physical examination showed bilateral nontender swelling of the arms and a 1 cm shorter left leg compared to the right. On laboratory investigation, her complete blood count and renal function tests were in the normal range. Serum calcium and alkaline phosphatase levels were also normal.

**Figure 1:**
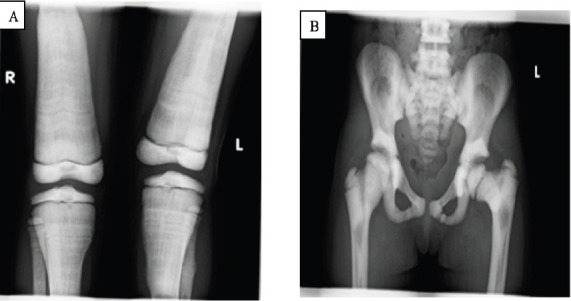
(A) Radiographs of the bilateral knee demonstrating metadiaphyseal widening with vertical and horizontal striations as well as loss of corticomedullary differentiation. (B) Pelvic x-ray demonstrating diffuse sclerosis with some spared normal-density bone indicating a “bone in bone” appearance, and lower lumbar vertebrae showing “Rugger jersey spine” or “sandwich vertebrae”

**Figure 2:**
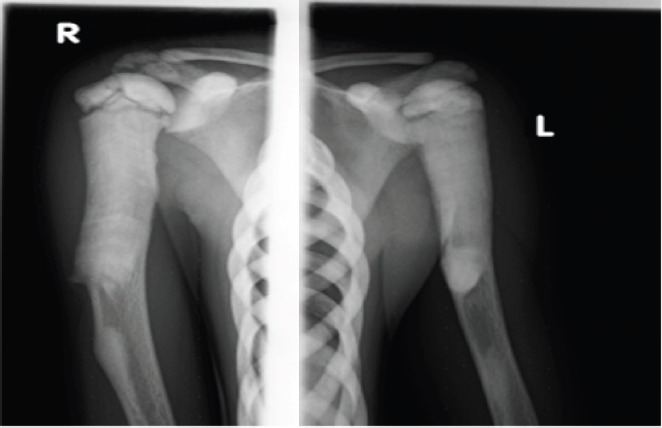
Radiograph of bilateral shoulders and upper arms showing increased bone density involving the medulla and cortex of the diaphysis, metaphysis, and epiphysis with loss of cortical and medullary differentiation

**Figure 3:**
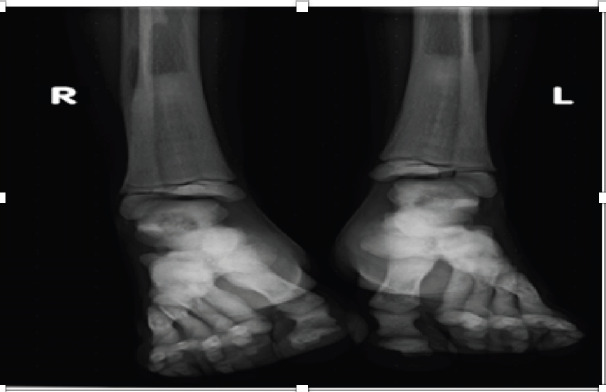
Bilateral frontal foot X-ray showing distal tibiofibular, tarsal, metatarsal, and phalangeal sclerosis

The plain radiographs of the long bones showed homogenously increased bone density involving the diaphysis, metaphysis, and epiphysis (Figures 1-3). There was also a loss of corticomedullary differentiation and vertical and horizontal striations. There was a widening of the metaphysis, also known as the “Erlenmeyer flask” deformity (Figure 1A). A pelvic x-ray revealed diffuse sclerosis with some spared normal-density bone, indicating a “bone in bone” appearance (Figure 1B). In the included lower lumbar vertebrae, the vertebral end plates have increased density, resulting in a “Rugger jersey spine” or “sandwich vertebrae” with involvement of the transverse process of the lower lumbar vertebrae (Figure 1B). There was also involvement of the tarsal, metatarsal, and phalangeal bones (Figure 3).

## Discussion

Osteopetrosis is a group of rare inherited metabolic bone diseases with variable clinical severity (1-3). Among the various forms of the disease, the malignant autosomal recessive type is the severe form, which is usually fatal in early childhood, and the autosomal dominant type is usually asymptomatic until adulthood (1,3).

In the ARO type, patients present within a few months of delivery (1) with signs and symptoms related to bone marrow failure, cranial neuropathies, and frequent pathologic fractures (1,4). The IAO recessive form occurs in the first decade of life, and its symptoms are similar to that of the ARO type but are less severe. Patients with ADO usually remain asymptomatic until adulthood. In type I, isolated osteosclerotic thickening of the cranial vault and cranial neuropathies usually occur, and there is no increased risk of fracture, whereas, in type II, patients usually develop anemia, pathological fractures, or early-onset osteoarthritis in adulthood (1).

In the present case, the patient's age (first decade of life), history of fractures, and imaging findings are consistent with ADO and IAO forms. However, ADO is more prevalent (1,2,4). Once osteopetrosis is diagnosed, genetic testing is recommended to assess the presence of a mutation, as subtypes respond differently to treatments and differ in prognosis and risk of recurrence (3). Plain radiographs will show diffuse sclerosis of the bone, giving it a “marble bone” appearance (1). “Erlenmeyer flask” deformity can be present at the metaphysis of long bones, particularly the distal femur, due to club-shaped flaring of the metaphysis of long bone with cortical thinning (2). A “bone-in-bone” parallel band of dense bones can be seen and is usually prominent in the pelvis, long bones, phalanges, and spine (1, 2). Furthermore, a “Rugger jersey spine” or “sandwich vertebrae” appearance is seen in the axial skeleton (vertebrae), where the endplates have excessive thickening with normal-appearing vertebral midbody between superior and inferior endplates (1-3). The “sandwich vertebrae”, the “bone-in-bone” appearance, and the “Rugger-jersey spine” are more frequently found in ADO type II (3). A dedicated CT or MRI scan can be used to evaluate the patency of the optic canal and can help confirm entrapment of the optic nerve (2, 3). It is crucial to remember that even if patients do not have symptoms initially, they can have progressive visual loss. Our patient was lost to follow-up, and therefore, cross-sectional imaging was not performed.

Skeletal fluorosis is a competing differential diagnosis in endemic areas. However, the patient had no history of prolonged fluoride exposure, joint pain, or imaging findings such as increased cancellous bone, connective tissue calcification, osteophytes, or periosteal bone proliferation that would suggest this diagnosis. Blood dyscrasias such as myeloproliferative neoplasia, acute leukemia, and lymphoma were very unlikely because the patient had no signs, symptoms, or laboratory findings to support these diagnoses. Osteoblastic bone metastasis was excluded because the patient had no known primary malignancies, and the bone sclerosis was generalized in nature.

If osteopetrosis is not treated properly, complications, including osteomyelitis, increased intracranial pressure, cerebellar tonsil herniation, hydrocephalus, and pancytopenia can result (2). The treatment of osteopetrosis must be individualized (1). Interprofessional care and monitoring are the cornerstones of optimal care (1, 2), which is primarily supportive since there is no known cure (1).

Hematopoietic stem cell (HSC) bone marrow transplantation is performed for the malignant autosomal recessive form of osteopetrosis. For those unsuitable for bone marrow transplantation or as a bridging therapy, interferon-gamma can be used (1). As a second-line treatment, corticosteroids can be used in severe infantile osteopetrosis, for whom hematopoietic stem cell transplantation is inappropriate (2).

A routine ophthalmologic examination is required, and in some cases surgical decompression of the optic nerve is necessary. In addition, routine dental examinations are necessary to prevent complications such as osteomyelitis of the mandible. Fractures and arthritis are best treated by orthopedic surgeons as their treatment is often associated with complications (1, 2). Hypocalcemia and secondary hypoparathyroidism can be treated with calcium and vitamin D, and red blood cell transfusions can be used to treat anemia (2).

In summary, osteopetrosis is a rare inherited bone disease of variable severity due to underlying genetic disorders. Patients can develop complications such as pathologic fractures and cranial neuropathies at a young age. It is important to perform appropriate imaging studies and refer patients for assessment and treatment by a multidisciplinary team.
